# Efficiency dynamics among onion growers in Maharashtra: a comparative analysis of drip irrigation adopters and non-adopters

**DOI:** 10.1186/s12870-024-04875-2

**Published:** 2024-04-03

**Authors:** Rajiv B. Kale, Abhishek D. Gavhane, Vishal S. Thorat, S. S. Gadge, Sagar M. Wayal, Shivam Y. Gaikwad, Sharadveer Singh, Kiran S. Khandagale, Rohini Bhat, Vijay Mahajan

**Affiliations:** 1 ICAR-Directorate of Onion and Garlic Research, Pune, Maharashtra India; 2 Navsari Agricultural University, Navsari, Gujrat India

**Keywords:** *Allium cepa*, Technical efficiency, Drip irrigation, Yield gap, Input efficiency, Constraints

## Abstract

**Background:**

Onions are economically and nutritionally important vegetable crops. Despite advances in technology and acreage, Indian onion growers face challenges in realizing their full productivity potential. This study examines the technical efficiency of onion growers, the factors influencing it, and the constraints faced by those adopting drip irrigation in the Ghod river basin of western Maharashtra. A sample of 480 farmers including those practicing drip irrigation and those not practicing it, was selected from Junnar, Shirur, Parner, and Shrigonda blocks of the basin. The primary data was collected through semi-structured interviews. Analytical tools such as the Cobb-Douglas production function (represents technological relationship between multiple inputs and the resulting output), a single-stage stochastic frontier model, the Tobit model, and descriptive statistics were used to assess the technical efficiency of onion production at the farm level.

**Results:**

According to the maximum likelihood estimates of the stochastic frontier analysis, drip adopters exhibited a mean technical efficiency of 92%, while for non-adopters it was 65%. It indicates that the use of drip irrigation technology is associated with higher technical efficiency. The association of technical efficiency and socio-economic characters of households showed that education, extension contacts, social participation, and use of information sources had a positive influence on technical efficiency, while family size had a negative influence on the drip irrigation adopters. For non-drip adopters, significant positive effects were observed for landholding, extension contact, and information source use. The major constraints faced by drip system adopters included a lack of knowledge about the proper operating techniques for drip systems and the cost of maintenance.

**Conclusion:**

The differences with inputs associated with two irrigation methods showed that the response of inputs to increase onion yield is greater for farmers who use drip irrigation than for farmers who do not, and are a result of the large differences in the technical efficiencies. These inefficiencies and other limitations following the introduction of drip irrigation, such as lack of knowledge about the proper operations, need to be addressed through tailored training for farmers and further interventions.

**Supplementary Information:**

The online version contains supplementary material available at 10.1186/s12870-024-04875-2.

## Introduction

Vegetable crops play a pivotal role in sustaining livelihoods, alleviating poverty, and promoting economic growth in rural communities [1–4]. Compared to other food crops, vegetable farming requires more economic activities and more inputs, making it a very intensive agricultural activity. For example, vegetable cultivation requires frequent irrigation, special fertilizers, and rigorous pest management, resulting in a more complex and resource-intensive cultivation process than other staple food crops. Onion (*Allium cepa*) occupies a significant position among vegetables as they are in constant demand, both in the domestic and international markets, due to their important role in the culinary practices and nutritional value [5, 6]. Moreover, the value addition and processing of onions in agro-based industries promote economic growth in rural areas [7]. The global production of onions reached 107 million tonnes (including shallots) in 2021, covering an area of 5.78 million hectares [5–8]. India is the leading onion-producing nation with 30.19 million tonnes of production in 2022-23, followed by China and Egypt [8]. Onion holds paramount importance in India’s agricultural landscape along with other vegetable crops, where 17.40 lakh hectares of onion cultivation was done in 2022-23. Onion production in India has increased from 6 million tonnes in 2001 to 30.19 million tonnes in 2022-23, which is a 5.8-fold increase [8–10]. The area under onion cultivation also witnessed a threefold increase from 2001 to 2022. Despite a substantial increase in the area under cultivation, onion productivity has only increased 1.64-fold, reaching 17.85 metric tonnes per hectare in 2022 from 10.59 metric tonnes per hectare in 2001 [8–10]. Here, effective management of resources such as water and nutrients are proving to be a critical factor in ensuring sustainable onion productivity. Water is the most valuable and indispensable resource for agriculture [11–13]. Prudent utilization of water resources is becoming a pressing concern for farmers worldwide [14–16]. Conventional irrigation methods in onion farming are often associated with inefficient water use and lower yields, necessitating a transition to more efficient irrigation practices [17–20]. In this context, the adoption of drip irrigation technology in onion cultivation emerges as a promising solution to address water use inefficiencies while optimizing crop yields and qualities [21–23]. Maharashtra is the leading onion-growing state in India, contributing 39.28% of the country’s onion production and 43.00% of the area under onion cultivation. The Ghod river basin is one of the prominent onion-growing areas in western Maharashtra [24, 25]. The secure water availability of the Ghod river and its well-developed water infrastructure have catalyzed significant growth in industries and the service sector in addition to the predominant agricultural activities in the region [24]. Proximity to major metropolitan areas like Pune and Mumbai has intensified agriculture, implying the need for efficient allocation of production resources to sustain productivity over the dominance of water by other activities, along with labour shortages, expensive inputs, and limited land holdings. Over the past two decades, the popularity of efficient water management technologies, including the drip method of irrigation has increased in the region [25, 26]. The intensification of agriculture, especially vegetables, has increased significantly with the adoption of the drip irrigation method. However, as farmers have limited sources for new investments in modern agricultural technologies [27], drip irrigation technology needs to be efficient in several aspects to become a profitable economic factor [28]. In addition to rationalizing the use of water and increasing its efficiency, the introduction of drip irrigation technology should also help to further improve the performance of other agricultural inputs. Internal inefficiencies of farmers in the use of inputs and technologies are a greater concern behind low productivity [29, 30] than the availability of the inputs. To increase production, expanding the area under cultivation is a challenge [31, 32], but the opportunity lies in the refinement of existing production technologies and the efficiency of available farm resources. The inefficiency of farmers may also result from lack of experience, illiteracy and various other socio-economic factors [33]. If farmers are technically inefficient, there is an opportunity to increase productivity by using better agricultural extension services and technological interventions. For this reason, it is important to assess the current efficiency of farmers in resource utilization for crop production, especially in irrigation methods [34]. It is equally important to examine the principal factors affecting farmers’ production efficiency, including the farmer’s socioeconomic variables [33, 35]. This study addresses fundamental questions regarding onion cultivation in the Ghod river basin: (i) are there differences in technical efficiency among onion growers depending on which irrigation method they use, and (ii) are the socioeconomic characteristics of those who use drip irrigation and those who do not use, it influences the technical efficiency of onion cultivation?

## Methods

### Study area

**Fig. 1:**
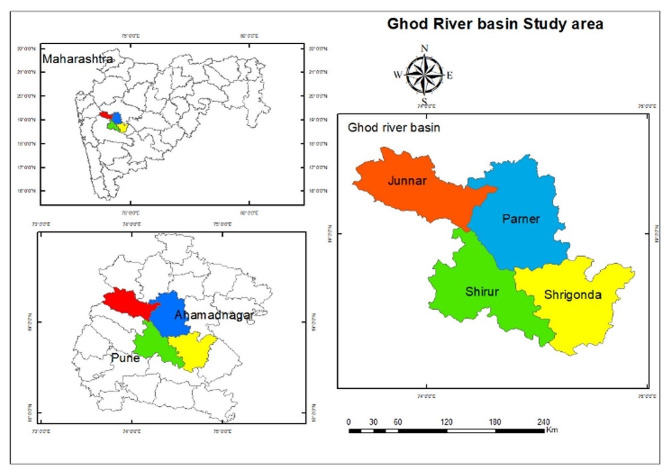
Location map of the study area

The study was carried out in the Ghod River basin in four blocks, namely Junnar and Shirur blocks in Pune district and Parner and Shrigonda blocks in Ahmednagar district of Maharashtra state, India (Fig. 1). Between 18°30′35″–19°24′55″N and 73°31′38″–74°43′53″E, on the eastern side of the Sahyadri mountain range in western Maharashtra. The altitude ranges from 494 to 1471 m above sea level, the average annual rainfall is about 741 mm, and the average temperature range is 24.88 °C [36]. The cropping patterns in the catchment area are very diverse due to the different geographical conditions, soil types, rainfall patterns, and availability of irrigation sources. Bajra, soybean, onion, groundnut and mung bean are usually grown during the *kharif* season [25]. In the absence of alternate irrigation sources, crops such as sorghum and chickpeas are grown, the success of which depends on rainfall and residual moisture. Similarly, in areas where irrigation projects are in operation, sugarcane, onions and fruits are mostly grown [25]. The cropping pattern is linked with water availability, market trends, and the availability of nearby markets.

### Sampling techniques

Respondent farmers were selected using a multi-stage random sampling technique. Data was collected through personal interviews using a pre-tested, semi-structured questionnaire during the late *kharif* and *rabi* season of 2022–2023 (supplementary file 1). Four blocks out of eight blocks were selected randomly from the Ghod river basin. Further from each block, seven villages were randomly selected for data collection. Respondents were randomly selected from a total of 28 villages based on the condition that he or she was an onion-growing farmer, irrespective of the size of their land holdings. A detailed sampling plan is given in Table 1. Half of the farmers from each block were adopters of drip irrigation systems, and the rest were non-adopters, i.e. they were using the traditional flat bed and flood irrigation method.

**Table 1:** Sampling Plan of the study

District	Block		Villages							Total
			1	2	3	4	5	6	7	
Pune	Junnar	Drip adopters	8	11	7	9	7	10	8	60
		Non adopters	11	6	11	8	8	9	7	60
	Shirur	Drip adopters	8	7	8	12	10	7	8	60
		Non adopters	10	11	6	7	7	8	11	60
Ahmednagar	Parner	Drip adopters	7	8	11	8	10	8	8	60
		Non adopters	8	12	8	7	10	8	7	60
	Shrigonda	Drip adopters	9	8	7	9	9	7	11	60
		Non adopters	10	8	6	7	11	8	10	60

*N* = 480

#### Estimation of technical efficiencies

In 1957, Farrel presented approaches for measuring efficiency [37]. Technical efficiency is measured as the ratio between the actual output of a farm to its own maximum possible frontier output for a given level of inputs and the chosen technology [38]. These approaches have been further extended by various researchers [39–41], who estimated a deterministic parametric frontier by specifying a homogeneous Cobb-Douglas production function. Although the Cobb-Douglas function is less flexible and simpler, it meets the self-duality criterion, which enables the analysis of technical efficiency. However, these approaches ignore the fact that the performance of a farm is influenced by exogenous factors such as weather, which are beyond the control of the farmer [42–48]. The stochastic frontier model takes into consideration the influence of uncontrollable exogenous shocks in the estimation process. The Cobb-Douglas model has been widely used in the above-mentioned empirical studies, particularly in developing countries to assess farm efficiency. The general form of the stochastic frontier function model with unobserved heterogeneity estimated is.

1 $${Y_i} = f\left( {{X_i},\,\beta } \right)\,exp\left( {{V_i} - \,{U_i}} \right)\,\,\,\,\,\,\,i = 1,2,3 \ldots .,n$$

Where: Yi = Onion output, i = the i^th^ farmer in the sample, X_i_ = a vector of inputs used by the i^th^ farmer, β = a vector of unknown parameters, Vi = a random variable which is assumed to be normally and independently distributed, and Ui = farm-specific technical inefficiency in production and nonnegative random variable. The lack of sensitivity analysis is acknowledged, as the strength of our methodology lies in the meticulous assessment of potential biases during data collection. The robustness of our findings is confirmed by their consistency in numerous statistical tests as well as the reliability of the underlying data. The computer programme FRONTIER version 4.1c was used for this study [45]. The farm level technical efficiency estimated in the study through Cobb–Douglas production form of stochastic frontier production can be stated as:

2 $$\begin{array}{l}Ln\,{Y_i} = \,{\beta _0} + \,{\beta _1}ln{X_1} + \,{\beta _2}ln{X_2} + \,{\beta _3}ln{X_3} + \,{\beta _4}ln{X_4}\\+ \,{\beta _5}ln{X_5} + \,{\beta _6}ln{X_6} + \,{\beta _7}ln{X_7} + \,{V_i} - \,{U_i}\,\,\,\,\,\,\,i = 1,2,\,3, \ldots ,240\end{array}$$

Where,

- Ln = Natural logarithm,
- Yi = Gross production of onion of i^th^ farmer (tonnes/ha).
- β_0_, β_1_,…β_7_ = Parameter to be estimated,
- X_1_ = Human Labour (man days/ ha).
- X_2_ = Machine labour (hours).
- X_3_ = Seed (Kg/ha).
- X_4_ = FYM (tonnes/ha).
- X_5_ = Fertilizer (Kg/ha).
- X_6_ = Number of spray (nos).
- X_7_ = Irrigation water applied (hacm).
- V_i_ = Random error having zero mean which is associated with zero factors which are not under the control of the farmer.
- U_i_ = One-sided inefficiency component.

Table 2 shows the details of explanatory variables and expected sign used in estimation of efficiency model.

**Table 2:** Explanatory variables for estimation of efficiencies and hypothesized sign

Sr. No.	Factor	Unit	Description	Expected sign
1	Human labour	Man days/hectare	Constitutes the cumulative effort invested in various pre-harvest to harvest activities, such as applying fertilizer, irrigation, sowing, transplanting, weeding, and harvesting. These tasks are measured in man-days per hectare to determine the total weighted labour (man equivalent) in person-hours, using a standard conversion factor. Given that labour plays a pivotal role in agricultural output, an abundance of domestic and other labour enables farmers to promptly implement essential crop husbandry techniques.	+/-
2	Machine labour	Hours/hectare	Machine working hours are the number of farm machinery hours required on one hectare of land, from initial tillage to the preparation of soil for nursery raising, transplanting, and other relevant field operations. The operating costs, labour costs, and fuel costs were considered to standardize the per-hour rate of machine use.	+/-
3	Seed	Kilogram/hectare	Seed is a prime input to govern the success of farm operations. It is hypothesized that seed quantity governs the seeding density and thereby may impact the yield positively or negatively.	+/-
4	FYM	Tonnes/hectare	FYM used in crop production is hypothesized to have a positive impact on soil properties, nutrient uptake, micronutrient status, and microbial status.	+
5	Fertilizer	Kilogram/hectare	The objective behind using fertilizer is to enhance crop output, yet excessive application can lead to reduced yields or even crop failure. Proper use of chemical fertilizers can substantially elevate production levels and improve farmer efficiency, resulting in positive outcomes.	+/-
6	Crop protection sprays	Numbers	It accounts for sprays taken for control of both fungal diseases and pests. Pesticide sprays are hypothesized to positively impact crop yield by minimizing the failure risk associated with different crop diseases and damaging pests.	+
7	Irrigation Water	Hectare centimeter /hectare	Generally, water is considered a growth and yield-limiting factor that governs crop yield. Both excess and less irrigation limit crop growth by affecting root functions and nutrient flow.	+/-

#### Tobit model and determinants

Measuring technical efficiency may not be an end in itself, but it underlines the importance of determining the factors that influence technical efficiency. Efficiency can only be improved if the determinants of efficiency can be identified. The estimates of technical efficiency range from 0 to 1, indicating that the dependent variable is limited. The Tobit model can manage this distribution of efficiency estimates, providing results that can guide policies to improve performance. This model is known as truncated or censored regression models having predicted errors that are not equal to zero. As a result, estimating technical efficiency estimates using ordinary least squares (OLS) regression would produce a biased parameter estimate [49]. The technical efficiency estimates derived from the stochastic frontier model were subjected to regression analysis with farm-specific explanatory variables using the two-sided Tobit model. The Tobit model was applied to find out factors that may affect farm technical inefficiency. Adopting this model remains the best approach because efficiency scores are the truncated variable that ranges between 0 and 1. The model’s theoretical formula is as follows:

$${\mu }_{i}^{*}={x}_{i}^{{\prime }}\beta +{u}_{i} \,\,\,\,\,\,\,\,\,\,i=\text{1,2},\dots \dots .,n $$

$$\it \tt \it \it \it \normalsize \large \it \it {\mu }_{i}={\mu }_{i}^{*}\,\,\,\,\,\,\,\,if\,\,\,\,\, {\mu }_{i}^{*} <0$$

$${\mu }_{i}=0, otherwise$$

Where ${u_i} \sim N\left( {0,{\sigma ^2}} \right),\,{x_i}\,and\,\beta$ are vectors of explanatory variables and unknown parameters, respectively. The ${\mu }_{i}^{*}$ is latent variable and ${\mu }_{i}$ is farm level technical efficiency score.

### Factors associated with technical efficiency as an independent variable

After a comprehensive review of the existing literature [47, 48, 50–58] and a contextual analysis of the study area, socioeconomic and institutional factors assumed to influence technical efficiency were selected as follows:

1. **Age**: The age of the household head is assumed hypothesized to reflect the farmer’s efficiency in managing agricultural enterprises. It can either have a positive or negative impact.
2. **Education Level of the Household Head**: Farmers are anticipated to exhibit enhanced management skills through formal education, which should have a positive effect on technical efficiency.
3. **Family Size**: Given the importance of the family as a labour resource in rural settings, larger households are expected to have an advantage in the efficient use of labour, especially during peak cropping seasons. However, the efficient handling of this force ultimately derives the impact.
4. **Experience in Farming**: The number of years of farming experience is directly related to the farmer’s experience in onion production and should have a positive impact on technical efficiency.
5. **Land Holding**: It is posited that land ownership has an impact on economies of scale and at the same time brings advantages and disadvantages in terms of sole supervision and management.
6. **Extension Contact**: It is expected that the contact with extension, represented by an index score based on interactions with different extension services, positively influences efficiency.
7. **Social Participation**: Active participation in social activities is assumed to enhance leadership and managerial qualities, positively affecting efficiency.
8. **Information Source Use**: An index score reflecting the utilization of diverse information sources by individual farmers to access information on onion cultivation technologies is considered a factor influencing efficiency positively.

The technical efficiency of each individual farm was worked out using the formula:

4 $$TE\, = \,{Y_i}/{Y_i}* $$

Where, Yi* is the frontier yield and Yi is the actual yield. Yi is the actual production of onion by i^th^ respondent in tonnes per hectare, Y_i_* is the potential production of onion by i^th^ respondent from the stochastic frontier model.

The difference pertaining to the actual and potential production of onions signifies the yield gap, which depicts the amount that can be added to the yield sum with maximal efficient use of inputs.

5 $${\rm{Yield \,gap}}\,{\rm{ = }}\,{\rm{(}}{{\rm{Y}}_{\rm{i}}}^{\rm{*}}{\rm{ - }}\,{{\rm{Y}}_{\rm{i}}}{\rm{) = }}\,{\rm{Potential}}\,{\rm{production}}\,{\rm{-}}\,{\rm{Actual}}\,{\rm{production}}{\rm{.}}$$

## Results

### Descriptive analysis

The synopsis of socio-economic profile and inputs employed by onion growers in the study revealed that those who used drip irrigation techniques produced an average yield of 36.23 tonnes/ha, which was higher than that of non-adopters, who produced an average yield of 30.13 tonnes/ha (Table 3). Notably, the average farming experience of drip adopters was 26.67 years lower when compared to the non-adopters (30.06 years). Compared to non-adopters, who reported an average yearly income of INR 288,270.42, adopters reported an income of INR 504,135.8 which was significantly greater.

**Table 3:** Socio-economic characteristics of onion producer and summary of Inputs and production

Socio-economic variable	Drip adopters		Non-adopters	
	Average	Std deviation	Average	Std deviation
Age of respondent	42.64	9.24	45.63	11.11
Educational years	11.24	3.16	9.61	3.66
Family size (Number)	6.23	2.60	6.30	2.63
Land holding (ha)	1.41	1.02	1.34	0.59
Family income (INR)	504,195.80	366,634.77	288,270.42	139,989.70
Experience of farming (years)	26.67	10.36	30.06	12.27
Extension contacts	0.36	0.12	0.28	0.11
Social participation	0.60	0.11	0.56	0.14
Information source use	0.67	0.08	0.72	0.11
Human labour (man days/ha)	195.17	4.98	238.58	9.02
Machine labour (hours/ha)	19.74	2.14	20.1	2.25
Seed (kg/ha)	6.95	1.93	8.86	1.40
FYM (tons/ha)	5.30	1.48	4.66	1.28
Fertilizer (kg/ha)	647.71	159.78	503.13	66.6
No of spray (number/ha)	7.06	1.26	8.92	1.09
Irrigation (hacm/ha)	58.73	10.01	81.07	11.97
Yield (tonnes/ha)	36.23	5.25	30.13	5.36
N	240		240	

The study also revealed that non-adopters demonstrated a greater utilization of human labour, employing an average of 238.58 man-days/ha, whereas adopters used a lesser amount at 195.17 man-days/ha. Similar to this, adopters utilized just 58.73 hacm of irrigation water per hectare for onion production, whereas non-adopters used an average of 81.07 hacm. Adopters applied an average of 647.71 kg of fertilizer per hectare, which was more than that of non-adopters, who only applied 503.13 kg of fertilizer per hectare to nourish their onion crops. 

### Econometric analysis

#### Maximum likelihood estimates of stochastic frontier model for onion crop production

The parameters of the stochastic production frontier model were determined using maximum likelihood estimation with the Frontier 4.1 computer program. The findings of the stochastic production function for onion production provide a useful insight into the factors that determine onion farming output. Each variable’s coefficient reflects the magnitude and direction of its impact on onion output. Cobb-Douglas’ production function estimation depicts estimates of the stochastic frontier production function from drip adopter and non-adopter onion producers in the Ghod river basin area (Table 4).

**Table 4:** Maximum likelihood estimates of stochastic frontier model for onion crop production

Variable	Parameter	Adopters of Drip			Non adopters of drip		
		Coefficient	standard error	*t*-ratio	Coefficient	standard error	*t*-ratio
Constant	β_0_	2.168**	0.944	2.297	3.629*	1.293	2.805
Ln (Human labour)	β_1_	-0.288***	0.169	-1.706	-0.141	0.202	-0.699
Ln (Machine labour)	β_2_	0.028	0.038	0.731	-0.129***	0.078	-1.647
Ln (Seed)	β_3_	0.127*	0.025	4.991	0.061	0.091	0.673
Ln (FYM)	β_4_	0.042***	0.023	1.833	0.115***	0.064	1.783
Ln (Fertilizer)	β_5_	0.341*	0.034	10.011	0.105	0.135	0.781
Ln (No. of sprays)	β_6_	0.042***	0.024	1.727	0.100	0.125	0.802
Ln (Irrigation water)	β_7_	0.086*	0.025	3.455	0.041	0.063	0.653
**sigma-squared**	**σ** ^**2**^	0.004*	**0.001**	**6.194**	0.013*	**0.001**	**10.604**
**gamma**	**γ**	0.632*	**0.136**	**4.644**	**0.7588**	**0.547**	**1.387**
**log likelihood function**		**357.87**			**177.15**		
**LR test of one-sided error**		**133.42**			**106.87**		
**Mean TE**		**0.92**			**0.65**		
**Yield gap**		**3.15**			**16.02**		
**Total sample size**		**240**			**240**		

(***, **, * indicates statistically significant at 10%, 5% and 1% level, respectively)

At a 1% probability level, the sigma (σ2 = 0.004 for adopters and 0.013 for non-adopters) is statistically significant demonstrating a good fit and validity of the distributional assumption of the composite error term. Gamma (γ) has a range between 0 and 1. If is close to 0, it indicates that deviations from the production frontier are largely caused by random noise, but a value close to unity for both adopters and non-adopters indicates that inefficiency is the cause of the majority of the deviations. As evidenced by gamma values of 0.759 for non-adopters and 0.632 for adopters, the research area’s irrigated onion production was found to have technological inefficiency impacts. As a result, 63.21% and 75.88% of deviations from the efficient frontier are caused by technical inefficiency in adopters and non-adopters, respectively. On the other hand, it suggests that variations in the producers’ technical efficiencies accounted for a significant percentage of the output variation observed in onions (the overall variation in output is attributable to the presence of production inefficiencies). The remaining 37% and 24% of the variation in output amongst onion growers who are drip adopters and non-adopters, respectively, can be attributed to mistakes made in data collection and aggregation, bad weather, the effects of pests and diseases, and unexpected circumstances. Within the drip irrigation cohort, all MLE coefficients, excluding machine labour, were found to be significant. The coefficients pertaining to seed, fertilizer, farmyard manure (FYM), number of sprays, and irrigation water exhibit statistical significance in the range of 1–10% level of significance, underscoring their substantial influence on onion production. These findings suggest that these variables are critical to the success of drip irrigation onion farming in the research area and that raising them further will boost onion yield. On the other hand, the non-adopters of the drip irrigation system segment showcase that the MLE coefficient corresponding to machine hours exhibits a negative sign but achieves statistical significance at the 10% level, indicating a distinctive impact. Furthermore, FYM coefficients maintain statistical significance at the 10% level among non-adopters. This indicates that in the flood irrigation setup, these two variables have some influence on the production of onions. According to the variations in the significant level of various inputs connected to the two irrigation methods, the reaction of inputs to increased onion output is larger in the drip irrigation system as compared to the conventional flood irrigation system.

#### Technical efficiency of onion farmers

The technical efficiency of surveyed individual farms was estimated through the utilization of maximum likelihood estimates derived from the Cobb-Douglas stochastic production function coefficients, as outlined in Table 5. According to the efficiency investigation the technical efficiency of non-adopter households ranged from 39 to 93%, with an average of 65%, whereas that of drip adopter onion households ranged from 78 to 99%, with a mean of 92%. In other words, households within the research area that employ drip irrigation or flood irrigation to grow onions generally experience output losses of 8% and 35%, respectively, due to technological inefficiencies. This indicates that the production loss due to technical inefficiency is higher for drip non-adopter onion farmers than for drip adopters. Analysis of the frequency distribution of technical efficiency among adopter farmers discloses that 2.08% of farmers operate within the efficiency range of 0.60–0.80, resulting in an average yield gap of 7.43 tonnes per hectare. The remaining 97.92% fall within the efficiency level greater than 0.80, with an average yield gap of only 3.06 tonnes per hectare. An overall average yield gap of 3.15 tonnes per hectare is reported for drip adopters. In contrast, Table 5 shows a distribution of non-adopter farmers: 0.42% work in the efficiency range of 0.20–0.40, 27.50% work in the range of 0.40–0.60, 69.17% work in the range of 0.60–0.80, and only 2.92% work at the maximum efficiency level of 0.80. Non-adopter farmers have a mean technical efficiency of 0.65 and a range of 0.39 to 0.93.

**Table 5:** Distribution of onion growers under different levels of technical efficiency

TE level	Drip (*N* = 240)				Flood (*N* = 240)			
	Frequency	Percent	TE	Yield gap (t/ha)	Frequency	Percent	TE	Yield gap (t/ha)
< 0.20	-	-	-	-	0	-	-	-
0.20–0.40	-	-	-	-	1	0.42	0.39	22.67
0.40–60	-	-	-	-	66	27.50	0.56	19.43
0.60–0.80	5	2.08	0.79	7.43	166	69.17	0.68	15.05
> 0.80	235	97.92	0.92	3.06	7	2.92	0.88	5.81
mean			0.92	3.15			0.65	16.02
max			0.99	9.67			0.93	26
min			0.78	0.37			0.39	3.24

In the non-adopter category, about 28% of farmers have technical efficiency below 0.60, which requires urgent attention. In comparison to onion growers who use drip irrigation, the mean yield gap that is, the difference between potential yield and actual output is bigger among non-drip irrigation producers (16.02 tonnes/ha) than it is among drip irrigation growers (3.15 tonnes/ha).

#### Technical efficiency and input use

The examination of input utilization patterns across different technical efficiency tiers of (Table 6) elucidates that drip adopter onion producers who, at the utmost technical efficiency level (> 80% efficiency), employed 195.27 man days per hectare of human labour, 19.76 h per hectare of machine labour, approximately 6.97 kg of seed per ha, 649.57 kg of fertilizer nutrients per ha, 5.34 tonnes of Farm Yard Manure (FYM) per ha, 7 crop protection sprays per ha, and 58.81 hectare-centimeters of irrigation per hectare of onion production.

**Table 6:** Input use of drip adopter onion growers under different levels of technical efficiency

TE level	Human Labour (Man days/ha)	Machine Labour (hours/ha)	Seed (kg/ha)	FYM (tons/ha)	Fertilizer (kg/ha)	No of spray (nos./ha)	Irrigation (hacm/ha)
< 0.20	-	-	-	-	-	-	-
0.20–0.40	-	-	-	-	-	-	-
0.40–60	-	-	-	-	-	-	-
0.60–0.80	190.60	19.00	6.20	3.60	560.00	6.20	55.00
> 0.80	195.27	19.76	6.97	5.34	649.57	7.08	58.81

Conversely, among non-adopters (Table 7), the subset exhibiting the highest efficiency deployed comparatively higher human labour (231.71 man-days), seed quantity (8.71 kg), crop protection sprays (8.71), and water (83.29 hectare-centimeters) per hectare for irrigating their onion crop.

**Table 7:** Input use of non-adopter onion growers under different levels of technical efficiency

TE level	Human Labour (Man days/ha)	Machine Labour (hours/ha)	Seed (kg/ha)	FYM (tons/ha)	Fertilizer (kg/ha)	No of spray (nos./ha)	Irrigation (hacm/ha)
< 0.20	-	-	-	-	-	-	-
0.20–0.40	225.00	20.00	7.00	3.00	400.00	8.00	80.00
0.40–60	237.88	20.24	8.35	4.18	475.76	8.50	77.32
0.60–0.80	239.18	19.95	9.08	4.86	514.46	9.10	82.48
> 0.80	232.71	22.14	8.71	4.71	507.14	8.71	83.29

Notably, a staggering 99.58% of non-adopter farmers employed a seed rate exceeding the recommended range (5–7 Kg/ha), thereby incurring elevated costs associated with this expensive input. Analogously, farmers exhibiting lower efficiency levels were predisposed to deploying more human labour, irrigation water, and less quantity of fertilizers, thereby amplifying inefficiencies in onion production and constricting the economical returns. This underscored that the misallocation of resources manifests as a hindrance to achieving optimal agricultural productivity.

#### Estimated potential yield under different levels of technical efficiency

**Fig. 2:**
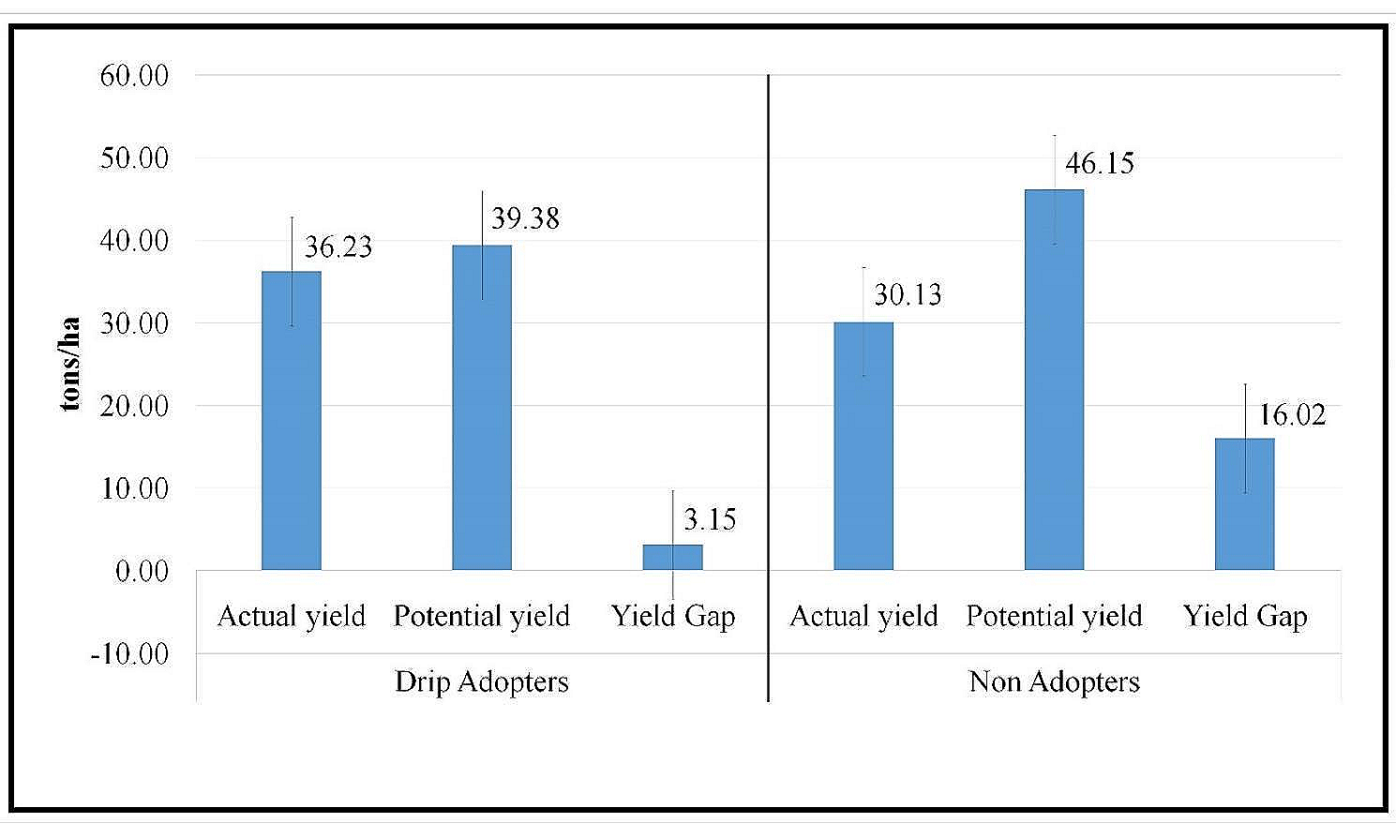
Actual and potential yields of drip adopters and non-adopters

The calculation of potential yield for each farmer was calculated, and the mean potential yield corresponding to distinct technical efficiency levels has been delineated in Table 8. The potential yield is obtained by dividing the actual yield by the technical efficiency of the farmer since efficiency is defined as the ratio of the actual yield attained to the greatest potential yield Based on the individual efficiency levels of the farmers, the potential yield of onions for drip-adopting households was estimated to be 39.38 tonnes per hectare, whereas for non-adopters, it was 46.18 tonnes per hectare (Fig. 2). There are two main causes for this higher potential output among drip irrigation system non-adopters. First off, in comparison to adopters, these onion growers are less technically efficient. It implies that there is a lot of room for adopting drip irrigation methods to boost current yield levels while still allocating resources in the most efficient manner possible. Second, non-adopter farmers might have more resources than adopters do; as a result, the yield level might be increased even further than drip-adopter farmers with the aid of careful planning and resource allocation.

**Table 8:** Mean technical efficiency, actual and potential yield under different levels of technical efficiencies

TE level	Drip(*N* = 240)			Flood(*N* = 240)		
	TE	Actual yield (t/ha)	Potential Yield (t/ha)	TE	Actual yield (t/ha)	Potential Yield (t/ha)
< 0.20	-	-	-	-	-	-
0.20–0.40	-	-	-	0.39	14.5	37.18
0.40–60	-	-	-	0.56	24.36	43.79
0.60–0.80	0.79	27.7	35.13	0.68	32.04	47.09
> 0.80	0.92	36.41	39.47	0.88	41.43	47.24
mean	0.92	36.23	39.38	0.65	30.13	46.18
max	0.99	51.5	57.36	0.93	47	54.20
min	0.78	24	29.14	0.39	14.5	35.73

Both adopters and non-adopters within the lower levels of technical efficiency exhibit higher yield gaps. Remarkably, among the least efficient farmers in the non-adopter category, where the average yield level currently stands at 14.5 tonnes per hectare, there exists the potential to nearly triple this output to 46.18 tonnes per hectare through judicious resource allocation by the farmers.

#### Factors affecting the technical efficiency of drip adopters and non-adopters for onion cultivation

The Tobit model regression analysis, as presented in Table 9, that the level of technical efficiency was negatively and significantly correlated with age, family size, and land holding size for drip-adopting farmers. In contrast, these adoptive farmers’ technical efficiencies were positively and significantly impacted by their education, farming experience, interactions with extension agents, social engagement, and usage of information sources. The results are plausible since increasing agricultural experience helps farmers deal with adversity, make the most use of existing resources, and make timely decisions on their farms. High extension contacts and social participation enable farmers to learn technical know-how, which contributes to increased productivity. Exposure to extension officials may encourage onion growers to use drip irrigation, increasing yield. Further, to use farm resources optimally, the results are in line with [59], who reported that farmers with higher education might have a greater capacity for the allocation of production resources.

**Table 9:** Estimates of the factors affecting the technical inefficiency model for onion production

Variable	Drip Adopters			Drip Non-adopters		
	Coefficient	Std. error	P>|t|	Coefficient	Std. err.	P>|t|
constant	0.6194	0.0255	0.0000	0.3840	0.0423	0.0000
Age of Respondent	-0.0003	0.0005	0.6420	-0.0001	0.0014	0.9330
Educational years	0.0018**	0.0008	0.0340	-0.0012	0.0012	0.3330
Family Size (Number)	-0.0019**	0.0007	0.0100	-0.0011	0.0014	0.4320
Land holding (ha)	-0.0001	0.0021	0.9700	0.0658***	0.0061	0.0000
Experience of farming (Years)	0.0007	0.0005	0.1370	0.0001	0.0012	0.9190
Extension contacts	0.2083***	0.0169	0.0000	0.1187**	0.0463	0.0110
Social Participation	0.1527***	0.0215	0.0000	0.2608***	0.0352	0.0000
Information Source use	0.1728***	0.0284	0.0000	0.0241	0.0425	0.5710
Log likelihood	510.88102			358.6267		
LRchi^2^	280.95			216.76		
Prob > chi^2^	0.0000			0.0000		
Pseudo R^2^	-0.3793			-0.4331		
N	240			240		

In the case of non-adopter farmers, the Tobit model unveiled a positive and significant association between land holding and technical efficiencies. Furthermore, extension contact and social participation displayed a positive and highly significant impact on technical efficiencies among non-adopter farmers.

#### Constraints faced by farmers while using drip irrigation systems in the study area

**Fig. 3:**
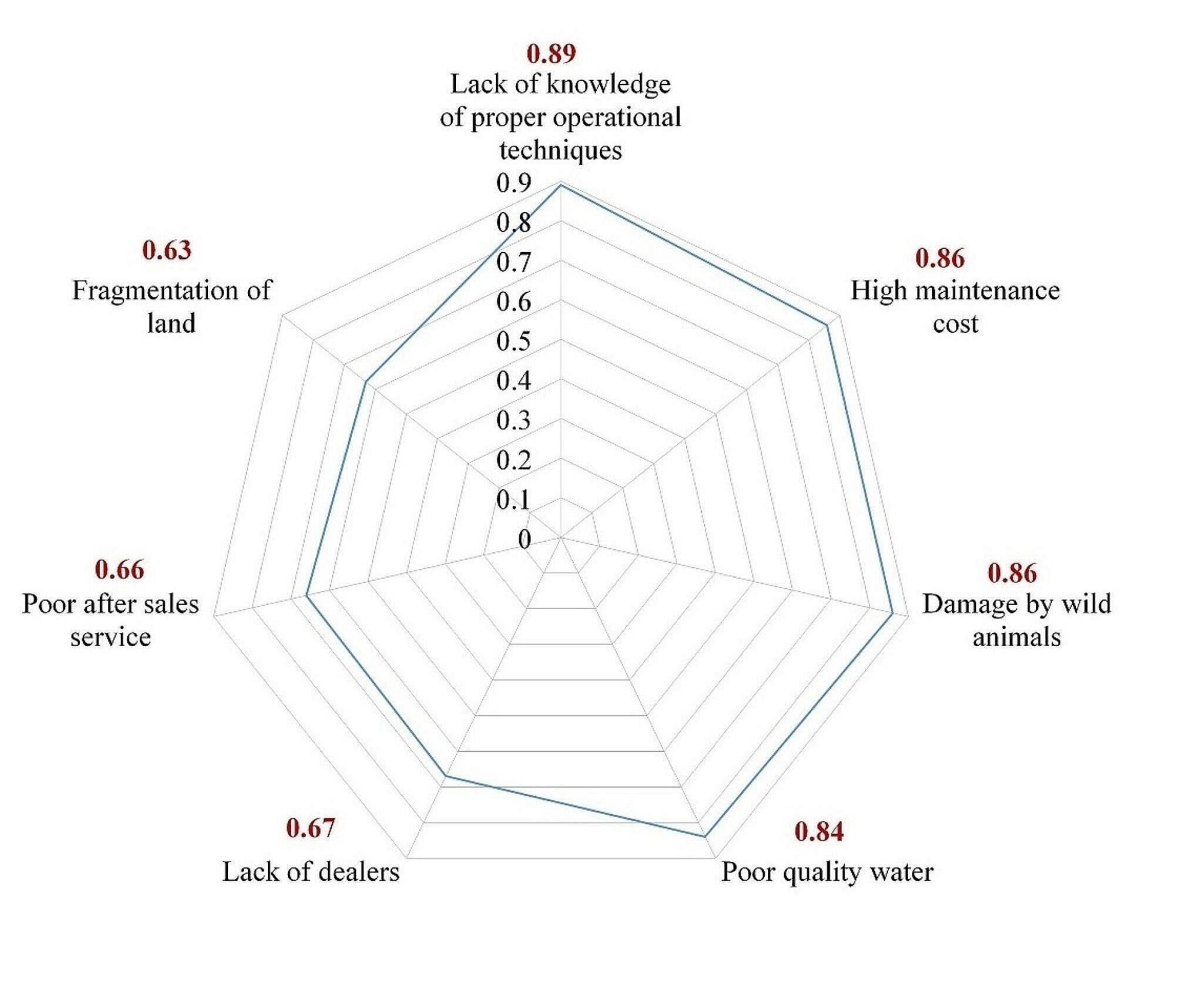
Constraints faced by farmers while using drip irrigation

Following the adoption of drip irrigation, farmers encountered significant challenges associated with operations and other factors. The assessment of such constraints in the study (Fig. 3) revealed that these constraints were primarily associated with the limited understanding of proper operational techniques (0.89) and the maintenance of micro-irrigation systems (0.86). The lack of knowledge regarding system operation posed a notable constraint, prompting many farmers to seek guidance from nearby dealers and fellow farmers in the vicinity. Among the hurdles faced, two prominent issues arose: damage to lateral lines caused by wild animals (0.86) and clogging (0.84) due to the usage of poor-quality water and fertigation practices.

A limited number of farmers identified poor after-sales service (0.66) and the lack of local dealers (0.67) as constraints. Another specific concern expressed by some farmers was the fragmentation of land (0.63). This apprehension primarily affected those who owned land parcels scattered across different locations. For these farmers, the fragmentation increased the complexity and time required for the installation and maintenance of drip irrigation systems.

## Discussion

With an emphasis on the use of drip irrigation techniques, the current study provides insightful information about the agricultural practices and socioeconomic dynamics of onion growers. The discovered variations between adopters and non-adopters indicate specific socio-economic elements that may impact their technical efficiency of onion growing in addition to illuminating the possible advantages of drip irrigation.

The economic implications of adopting drip irrigation are evident in the substantial difference in annual income between the two groups. The higher annual income range observed in adopter farmers underscores the potential base for their investment in the technology and support financial aid to the lower income category farmers for bridging the adoption decision. In contrast to non-adopters, adopters showed a lower average level of farming experience. This goes against research findings [60–65] which indicate that seasoned farmers are more technically proficient at farming due to their advanced abilities and increased confidence in the functionality of technology. Farmers with less expertise may be more inclined to experiment with and finally accept novel techniques that will transform their farming practices. The fact that non-adopters used more human labour suggests that drip irrigation is a labour-saving technique that lets adopters make the most of their labour resources. The higher income shown among adopters may be attributed to the decreased reliance on manual labour. The difference in irrigation water consumption is striking: adopters used only 58.73 hacm per hectare, while non-adopters used an average of 81.07 hacm. This suggests that adopters are more water-efficient, possibly as a result of drip irrigation’s ability to apply water in a targeted and controlled manner [66, 67]. Even while adopters applied fertilizer on average more frequently (647.71 kg/ha) than non-adopters (503.13 kg), the greater yield among adopters indicate a potential synergy between drip irrigation and fertilizer application practices in enhancing overall crop productivity [65, 66] but certainly show adopters’ inappropriate fertilizer management practices. In line with the findings by [60–65; 68, 69] regarding the relationship between farming experience and technology adoption, the adopters achieved a higher average yield, increased income, and more efficient resource utilization than non-adopters. This is indicative of the positive impact of drip irrigation on crop productivity.

The analysis, which is based on the stochastic frontier model’s maximum likelihood estimate, sheds light on important factors influencing onion production and emphasizes how technical inefficiency shapes output variations overall for both drip adopter and non-adopter onion producers (Table 4). The distributional assumption of the error component and the suitability of the model are both supported by the statistically significant sigma. This implies that the underlying dynamics of onion production in the area are well captured by the stochastic frontier production function. The gamma (γ) values also show that deviations from the production frontier are significantly influenced by technical inefficiency. The frequency of inefficiencies in the production process is highlighted by the comparatively high gamma values [60]. This could be attributed to factors such as suboptimal use of inputs like labour, fertilizers or pesticides. This suboptimal use is high among non-adopters of drip irrigation as compared to adopters explained through the relatively higher gamma value for non-adopters. The necessity for focused interventions to increase efficiency is implied by the finding that technical inefficiency is a significant factor influencing onion production in both adopters and non-adopters. Techniques to improve farmers’ understanding, encourage excellent agricultural practices, and ease the adoption of new technology are essential to resolve the inefficiencies. To further improve overall production efficiency, a comprehensive approach must address variables including pests, diseases, and unfavorable weather conditions that contribute to the remaining variation in onion output amongst producers. The influence of technology adoption on production efficiency is shown by the significant disparities in the proportion of deviations between adopters and non-adopters, which can be attributed to technical inefficiencies. This discrepancy implies that adopters of drip irrigation are in a better position than their non-adopter counterparts to minimize inefficiencies and optimize their production processes. For both adopters and non-adopters, a thorough analysis of the particular factors affecting onion yield under various irrigation techniques provides insightful information. The factors including seed, fertilizer, farmyard manure (FYM), number of sprays, and irrigation water highlights their crucial role in improving onion yield. The positive coefficient of irrigation water, fertilizer, crop protection sprays, seed, FYM, and irrigation water indicate that onion yield grows in sync with increament in each of these input factors.

The fact that the coefficient for human labour is negative indicates that both adopters and non-adopters in the area are using excessive and wasteful amounts of labour to produce onions. The coefficient for labour used in adopters’ production has a negative sign, indicating that adding more labour may not increase onion production. The result is statistically significant, as indicated by the related *P*-value, which suggests a relative drop of 0.29% with every additional unit of labour used. These results can be linked to inefficient human work and are not consistent with [68–72]. Harvesting and transplanting seedlings account for the majority of the human labour needed for onion production in the research area.

Because of the proximity to cities and industries, the labour force is primarily directed towards stable employment sources, which leads to inefficient skills regarding farm operations in the labours resourced for a little period. To redirect these workers for seasonal operations, they are also paid significantly more than the usual pay. In the case of non-adopter farmers, the negatively significant coefficient for machine labour suggests that increased machine hours may result in a marginal decline in onion production or a relative 0.13% decrease in production for every unit percent increase in machine labour. While non-adopters are assumed to form flat beds for flood irrigation, which requires comparatively more machinery use and relatively higher compaction of soil than the cultivation implying drip irrigation, adopters of drip irrigation for onion production require relatively less machinery for the general layout used for crop raising. These results create avenues for additional investigation to explore the causes of these results. This contradicts the results of previous research [49, 72].

On the other hand, the quantity of seeds used to grow the nursery shows a highly significant positive connection, suggesting that for drip adopter farmers, utilizing more seeds could have a somewhat favorable impact on onion yield [57]. Farmyard manure (FYM) has a significant positive coefficient (0.042 and 0.115) with 90% statistical significance for adopters and non-adopters, respectively. This means that applying more FYM can greatly increase onion production, so farmers should pay close attention to it. Fertilizer use has a very strong positive coefficient. However, the higher fertiliser use pattern in adopter farmers shows further scope in efficient management of this value input. Predominantly, during the survey, the farmers were observed to imply both the soil application and fertigation methods without considering the application efficiencies and crop needs. More research is necessary to ascertain the precise effect of fertilizer intake on the efficiency of onion production.

Increasing the number of crop protection spray significantly increases onion production, as evidenced by the positive coefficient and statistically significant *P*-value in the case of adopters. This finding supports previous reports [73] that highlight the significance of pest and disease management in onion farming. It was also discovered that the amount of irrigation water used to grow onion crops throughout the season was much higher for non-adopters but negligible for the drip adopters. This suggests that in the case of drip adopters, irrigation helped to improve onion production. This increase in onion yield highlights the possible advantages of effective irrigation techniques.

There is a clear difference between drip adopters (78–99%) and non-adopters (39–93%) in terms of observed technical efficiency ranges (Table 5). When it comes to technical efficiency, drip adopters routinely outperform non-adopters. This disparity in effectiveness highlights how the selected irrigation method affects the results of onion production. The technical efficiency frequency distribution between drip adopters and non-adopters reveals that most drip adopters (97.92%) function at an efficiency level higher than 0.80; only a small percentage (2.08%) operates in the 0.60–0.80 efficiency range, indicating that some farmers’ practices may need to be improved to get them closer to their production frontier. On the other hand, the technical efficiency distribution among non-adopters is characterised by a more widespread pattern. The efficiency threshold of 0.60 is not met by approximately 27.92% of non-adopters, indicating a severe need for interventions to enhance their production methods. The comparison of the mean yield gap between drip adopters and non-adopters highlights how important irrigation method adoption is to attaining agricultural output. Higher onion yields can be achieved by effective resource management and utilization, of which drip irrigation is a major contributor. Technical efficiency interventions must be tailored to the individual requirements of drip adopters as well as non-adopters.

In the first case, the focus should be on the small minority of farmers operating in the 0.60–0.80 efficiency range; in the later case, a larger-scale effort is needed to raise a sizable percentage of farmers operating below the 0.60 barrier. Generally, to get non-adopter farmers closer to their production frontier, interventions such as the adoption of better technology tools for cultivation, knowledge of best practices, and capacity building should be the primary focus. To improve total onion production efficiency in both irrigation systems, policymakers and researchers can investigate the mechanisms behind the variations in efficiency levels and pinpoint best practices from high-performing farmers. The input utilisation pattern of adopters and non-adopters, in conjunction with their corresponding technical efficiency categories (Tables 6 and 7), indicates that farmers with lower efficiency levels employ more labour, seeds, and crop protection sprays. Because of the inefficient use of production inputs and managerial choices, this validates their respective rankings in the efficiency categories [21, 67, 68, 74]. This highlights the significant opportunity to close the yield gap between actual and prospective by optimizing resource use in agriculture, particularly for non-adopter farmers with lower technical efficiency levels (Table 8). Construction of irrigation channels connecting beds to the water source is required for the conventional flood irrigation layout. This implies the usage of more tillage (machinery use) and labour. For these components, drip adopter farmers show marginal savings. There was a noticeable difference in the input utilization between irrigation water and seed. When it comes to seeds, the irrigation technique itself explains the increased water consumption; higher seed rates may result from increased mortality in flat bed systems. Farmers used more seeds to compensate for mortality losses. The suitable conditions for seed germination are obvious with drip irrigation systems causing consumption of significantly smaller seed quantities. Furthermore, the slight increase in the quantity of crop protection sprays leads us to the conclusion that traditional flat-bed irrigation systems have relatively higher disease and pest conditions. Overall, it is certain that judicious resource utilization is a crucial factor in bolstering crop performance and improving in technical efficiencies of onion farming.

Understanding the various factors at work requires analyzing the factors that determine technological efficiency. The Tobit model’s results (Table 9) highlight the complex interactions between socio-personal factors that influence respondent onion growers’ technical efficiency.

The dynamic feature of generational differences in adopting agricultural innovations is revealed by the age factor. Similar to the findings of [49, 60, 68], the age of the household head was found to be a significant factor negatively affecting the technical efficiency of both drip irrigation method adopters and non-adopters. This suggests that older farmers are more reluctant to adopt than younger farmers, who are more likely to choose cutting-edge modern techniques over time-honored ones when growing onions.

The relationship between **education levels** and technical efficiency varied among adopters and non-adopters. Drip adopters demonstrated a positive association, implying that higher education equips individuals with better farm management skills [60]. This underscores the significance of education in adopting advanced technologies and understanding optimal input utilization. Conversely, non-adopters with higher education levels showed a negative association, suggesting a segment of educated farmers resistant to agricultural changes.

**Family size** emerged as a significant negative factor affecting technical efficiency for both adopters and non-adopters. This underscores the challenges associated with managing the workforce within larger family units. However, the nuanced nature of this impact hinges on the quantity and calibre of family members who work as farmers, which emphasizes the importance of taking these variables into account in addition to family size. These outcomes are consistent with the conclusions of [75, 76] and contradict those of [77].

**Farming experience** was found to be insignificant in affecting technical efficiency in both adopters and non-adopters. Experience in agriculture is generally linked to improved skill development and is essential for developing agricultural managerial competency through experiential learning [60]. These results run counter to [60–64]. This is comparable to farmers who have grown more adept at farming using the old methods and have grown resistant to using the new ones. On the other hand, less experienced farmers can more readily absorb modern technology and are less set in their ways than older ones, which leads to greater results.

Both drip adopter and non-adopter farmers benefited from good effects of **extension contact** on onion production efficiency, highlighting the crucial role it plays in maximizing agricultural techniques. According to this association, farmer involvement with extension agencies effectively mitigates inefficiencies in resource allocation and management decisions, leading to increased efficiency in onion production. The benefits of extension services may be ascribed to several things, including easier access to current agricultural knowledge, advice on best practices, and support in overcoming obstacles. Similar outcomes were documented by [78–80].

The farmers who responded showed a statistically significant positive correlation between their technical efficiency in growing onions and their **social participation**. This link can be ascribed to more chances to make institutional contacts and concurrent exposure to state-of-the-art agricultural technologies. Given that social participation and technical efficiency are positively correlated, networking and cooperative engagement within agricultural communities are critical for information dissemination, experience sharing, and technology adoption. Additional insights into the mechanics of this relationship may become apparent through further research into the particular methods of social engagement, such as farmer cooperatives or community projects.

Similarly, the impact of **information source use** on technical efficiency was observed to be positive in both respondent cohorts. These results are in line with the findings of [48–56]. A higher information source index indicates an extensive reach of literature and information content among farmers. It has helped with the improvisation in current cultivation practices and informed decision-making regarding the adoption of new technologies. Beyond the apparent positive association, exploring the nature of information sources, such as agricultural extension materials, news dailies, magazines, journals, or digital platforms, could provide nuanced insights into the diverse channels through which farmers access valuable information. This may unveil potential strategies for enhancing information dissemination and promoting effective knowledge transfer within agricultural communities.

The constraints faced by post-drip irrigation adoption highlight crucial considerations for improving the implementation of this technology in farmers’ fields. The major constraints found were a limited understanding of operational techniques and the high maintenance cost of micro-irrigation. Most of the farmers surveyed faced maintenance-related problems due to the quality of lateral lines and small components. Further, it can be considered as a subsequent result of the knowledge gap of proper operational techniques such as periodical acid wash, fertigation grade fertilizers and bio-solutions, water filtration, pressure systems etc. It suggests that to improve farmers’ knowledge and abilities, capacity building among them through specialized farmer training programs is necessary. In agricultural contexts, the requirement for protective measures and wildlife management tactics is justified by the serious threat that wild animals may cause harm to drip systems. Although after-sales service limits are not given as much attention, governments and supporting organizations should do more to help resolve these problems because there are fewer local dealers and greater upfront expenditures. Together, governments and agricultural stakeholders can address these barriers with targeted interventions and demand-driven strategies to help farmers sustainably adopt drip irrigation practices.

## Conclusion and policy implication

This study aimed to evaluate the technical efficiency of onion growers in the Ghod River basin area of Maharashtra state, India, both by adopting and not adopting drip irrigation technology, as well as by examining the factors that influence their technical effectiveness. The output level can be increased by 35% in the case of non-drip adopters and 8% in the case of drip adopters, considering the mean efficiency levels of 0.65 and 0.92. All MLE factors, excluding machine labour, were determined to be significant within the drip irrigation adopter cohort. With statistical significance in the range of 1–10%, the coefficients for seed, fertilizer, farmyard manure, number of sprays, and irrigation water demonstrate their substantial impact on onion output. On the other hand, the segment that did not implement drip irrigation shows that the two MLE coefficients related to machine hours and FYM were determined to be significant. The reaction of inputs to improved onion output is stronger in drip irrigation adopter farms than in non-adopter farms, as evidenced by the differences in the significant level of various inputs connected with the two irrigation techniques.

The Tobit model regression analysis revealed that the degree of technological efficiency was found to be negatively correlated with age, family size, and land-holding size among drip-adopting farmers. In contrast, these adoptive farmers’ technical efficiencies were positively and significantly impacted by their education, farming experience, interactions with extension agents, social engagement, and usage of information sources. The findings make sense because a greater level of farming experience aids farmers in overcoming obstacles, making the most of their resources, and making quick judgments about their operations. Farmers who participate in social media and have strong connections with extension agencies can acquire technical know-how, which helps them use resources as efficiently as possible.

The study’s assessment of the restrictions indicated that the maintenance of micro-irrigation systems (0.86) and a restricted comprehension of appropriate operating approaches (0.89) were the main sources of the constraints. One significant obstacle was the lack of knowledge about how the system worked, which caused many farmers to ask local merchants and other farmers for advice. In addition, two more problems that require care are lateral line damage from wild animals (0.86) and clogging (0.84) from using low-quality water and incorrect fertigation techniques.

In light of these findings, the following policy recommendations ought to be taken into account:

1. To induce onion growers to use drip irrigation technology, specific incentive programs must be introduced, with a focus on minimizing the significant technical inefficiencies found among non-adopters.
2. The creation of a specialized drip irrigation training manual with an emphasis on best practices for farmyard manure application, spray control, fertilizer application, seed selection, and irrigation water optimization for farmers.
3. Policy interventions should prioritize the enhancement of extension services, given the acknowledged positive and highly substantial effects that education, farming experience, extension contact, and social participation have on the technical efficiency of drip adopter farmers.
4. Increased investment for extension programmes for more regular and meaningful interactions between farmers and agricultural extension experts.
5. Individualized support plans should be developed to satisfy the needs of drip irrigation users while accounting for the fluctuating influence of inputs on onion yield. Subsidies, rewards, and technical assistance in areas like a seed, fertilizers, farmyard manure, and irrigation water as these align with established factors of technical efficiency should be prioritized for drip adopter farmers.

### Electronic supplementary material

Below is the link to the electronic supplementary material.

Supplementary Material 1

## Data Availability

The dataset supporting the conclusions of this article is included within the article.

## Abbreviations

%: Percentage
mm: Millimeter
ha: Hectare
Kg: Kilogram
Nos: Numbers
hacm: Hectare centimeter
t/ha: Tonnes per hectare
INR: Indian Rupees
TE: Technical Efficiency
Ln: Natural logarithm
FYM: Farmyard manure
<: Less than
>: Greater than
N: Sample size
max: Maximum
min: Minimum
P>|t|: Probability
MLE: Maximum likelihood estimation
