# Supplementary material for: Efficiency dynamics among onion growers in Maharashtra: a comparative analysis of drip irrigation adopters and non-adopters
Source: BMC Plant Biol. 2024 Apr 3;24:237. doi: 10.1186/s12870-024-04875-2 (PMC10988828; doi:10.1186/s12870-024-04875-2)
Supplement: Supplementary file 1 — Supplementary Material 1 [file 12870_2024_4875_MOESM1_ESM.docx]

**Supplementary file: 1** Survey questionnaire for assessment of drip irrigation method in Ghod river Basin

**Basic Information**

1. Name of Farmer-

2. Village - Taluka- District-

3. Mobile No.-

4. Age-

5. Gender-

6. Education-

7. Family size-

8. Farm income per annum-

9. Experience of farming-

10. Land holding (ha)-

11. Net sown area (ha)-

12. Gross cropped area (ha)-

**Farming related information**

1. Area under onion cultivation: Season (ha): *Kharif*- Late Kharif- Rabi-

2. Number of plots of Onion-

3. Previous Crops & cropping pattern-……………………………….

4. Types of Beds-☐ Ridge and Furrow ii. ☐ Broad bed furrow iii. ☐Flat bed

5. Type of Soil-

**Irrigation related information**

1. Source of Irrigation water- ☐Well ☐River ☐Canal ☐ Tube well
2. Net irrigated area- hectare
3. Gross irrigated area- hectare
4. Type of Pump- ☐ Diesel ☐ Electric Pump ☐ Solar panel
5. Total number of irrigations in day’s-
6. What is the irrigation interval in hour-
7. Total hour of irrigation given during crop season- /ha
8. Horse power and Manufacturing company of installed pump-
9. Discharge rate of pump- litter per hour (LPH)

**Drip Irrigation Technology** (To be filled by drip irrigation users only)

1. Area under drip in onion cultivation- hectare

2. Other crops under drip irrigation-

3. Type of filter used for drip irrigation-

4. From which year you are using drip irrigation method?

5. Length of pipe line from, irrigation source-

6. No. of beds and size-

7. No. of laterals/bed-

8. Length of bed/lateral-

9. Distance between drippers-

10. Discharge rate of Dripper-

11. Length of main line: No of lateral-

12. Thickness of lateral: mm

**Fertilizer management**

1. Which type of fertilizer you use? ☐ Organic ☐ Inorganic ☐ Both

2. Do you use fertigation method? ☐ Yes ☐ No

3. Nutrient Management (fertilizer & quantity):

i. Basal dose-

ii. Top dressing-

iii. Water soluble-

**Input use**

1. Seeds- kg/ hector of transplanting
2. Hired labour- days/ha in entire season
3. Machine labour- hours/ha in entire season
4. Family labour- days/ha in entire season
5. No. of pesticide sprays- /ha in entire season
6. Farm yard manure used- /ha in entire season
7. Total fertilizer used- /ha in entire season

**Output**

1. Yield- bags/acre or ha

2. Produce quality:

% Marketable Bulbs:

%A-grade bulbs:

%B-grade bulbs:

%C-grade bulbs:

% Unmarketable:

% Bolters:

% Doublers:

**Constrains faced by adopters while using drip method of irrigation** (To be filled by drip irrigation users only)

| SN | Particular | Highly Relevant | Relevant | Not Relevant |
| --- | --- | --- | --- | --- |
| 1 | Poor quality of irrigation water |  |  |  |
| 2 | High need/cost of maintenance in drip irrigation |  |  |  |
| 3 | Lack of knowledge or proper operational techniques |  |  |  |
| 4 | Lack of drip irrigation system dealers in area |  |  |  |
| 5 | Poor after sales service |  |  |  |
| 6 | Fragmentation of land |  |  |  |
| 12 | Damage by wild animals |  |  |  |

**Extension contacts**

| SN | Extension workers/ Agencies | Weekly | Fortnightly | Monthly | Yearly | Never |
| --- | --- | --- | --- | --- | --- | --- |
| 1 | Gram Sevak |  |  |  |  |  |
| 2 | Agricultural Officer |  |  |  |  |  |
| 3 | University |  |  |  |  |  |
| 4 | Visit to Agricultural research institute |  |  |  |  |  |
| 5 | Visit to Kisan Melas |  |  |  |  |  |
| 6 | NGO |  |  |  |  |  |
| 7 | Other |  |  |  |  |  |

**Social participation**

| SN | Organization | Member | Office  Bearer | Participation | | |
| --- | --- | --- | --- | --- | --- | --- |
|  |  |  |  | Always | Sometimes | Never |
| 1 | Panchayat |  |  |  |  |  |
| 2 | Co-operative society |  |  |  |  |  |
| 3 | Youth club |  |  |  |  |  |
| 4 | Labour organization |  |  |  |  |  |
| 5 | Socio- cultural  Organization |  |  |  |  |  |
| 6 | Farmer Groups/ SHG |  |  |  |  |  |
| 7 | Local Bodies |  |  |  |  |  |

**Information source use pattern**

| SN | Information Source | Always | Sometimes | Never |
| --- | --- | --- | --- | --- |
| 1 | Radio, television |  |  |  |
| 2 | Newspaper |  |  |  |
| 3 | Agricultural publication/ journals |  |  |  |
| 4 | Agricultural guide / dairy |  |  |  |
| 5 | Agricultural seminars/ workshops |  |  |  |
| 6 | Agricultural trainings |  |  |  |
| 7 | Agricultural exhibitions /Demonstrations |  |  |  |
| 8 | Personal contacts |  |  |  |
| 9 | Electronic media (mobile phones/internet) |  |  |  |
| 10 | Drip agency |  |  |  |
| 11 | Krishi Seva Kendra/ Private input Supplier |  |  |  |
